# Application of a nonrandomized stepped wedge design to evaluate an evidence-based quality improvement intervention: a proof of concept using simulated data on patient-centered medical homes

**DOI:** 10.1186/s12874-016-0244-x

**Published:** 2016-10-21

**Authors:** Alexis K. Huynh, Martin L. Lee, Melissa M. Farmer, Lisa V. Rubenstein

**Affiliations:** 1 VA Greater Los Angeles HSR&D Center for the Study of Healthcare Innovation, Implementation & Policy (CSHIIP), 16111 Plummer St, Bldg 25, North Hills, CA 91343 USA; 2 RAND Corporation, 1776 Main St, Santa Monica, CA 90401 USA; 3 UCLA Geffen School of Medicine, 10833 Le Conte Ave, Los Angeles, CA 90095 USA; 4 UCLA Fielding School of Public Health, 405 Hilgard Ave, Los Angeles, CA 90024 USA

**Keywords:** Electronic measures, Evaluation of evidence-based quality improvement intervention

## Abstract

**Background:**

Stepped wedge designs have gained recognition as a method for rigorously assessing implementation of evidence-based quality improvement interventions (QIIs) across multiple healthcare sites. In theory, this design uses random assignment of sites to successive QII implementation start dates based on a timeline determined by evaluators. However, in practice, QII timing is often controlled more by site readiness. We propose an alternate version of the stepped wedge design that does not assume the randomized timing of implementation while retaining the method’s analytic advantages and applying to a broader set of evaluations. To test the feasibility of a nonrandomized stepped wedge design, we developed simulated data on patient care experiences and on QII implementation that had the structures and features of the expected data from a planned QII. We then applied the design in anticipation of performing an actual QII evaluation.

**Methods:**

We used simulated data on 108,000 patients to model nonrandomized stepped wedge results from QII implementation across nine primary care sites over 12 quarters. The outcome we simulated was change in a single self-administered question on access to care used by Veterans Health Administration (VA), based in the United States, as part of its quarterly patient ratings of quality of care. Our main predictors were QII exposure and time. Based on study hypotheses, we assigned values of 4 to 11 % for improvement in access when sites were first exposed to implementation and 1 to 3 % improvement in each ensuing time period thereafter when sites continued with implementation. We included site-level (practice size) and respondent-level (gender, race/ethnicity) characteristics that might account for nonrandomized timing in site implementation of the QII. We analyzed the resulting data as a repeated cross-sectional model using HLM 7 with a three-level hierarchical data structure and an ordinal outcome. Levels in the data structure included patient ratings, timing of adoption of the QII, and primary care site.

**Results:**

We were able to demonstrate a statistically significant improvement in adoption of the QII, as postulated in our simulation. The linear time trend while sites were in the control state was not significant, also as expected in the real life scenario of the example QII.

**Conclusions:**

We concluded that the nonrandomized stepped wedge design was feasible within the parameters of our planned QII with its data structure and content. Our statistical approach may be applicable to similar evaluations.

## Background

Stepped wedge designs have gained recognition among healthcare evaluators over the past decade as an alternative to classical cluster randomized designs for evaluating the implementation and spread of evidence-based healthcare quality improvement interventions (QIIs). QII process aims to translate prior efficacy and effectiveness evidence into routine policy and practice. Ultimately, QIIs must be implemented through local organizational improvement efforts and adapted to local contexts [1, 2]. Randomized stepped wedge designs enable successive implementation in sites over time, and have recognized sample size [3] and logistical feasibility [4–6] advantages over classical cluster randomized designs. However, unlike more traditional research projects where researchers control the intervention process, the ultimate control over a QII rests with local personnel, their leaders, and organizations. In this situation, the abilities of researchers to randomly time implementation is in question, as shown in recent systematic reviews of stepped wedge designs [5, 7]. In the work presented here, we explore application of a nonrandomized stepped wedge design using simulated data, in preparation for evaluation of an actual multisite QII in the Veterans Health Administration (VA), the largest integrated health care system in the United States. We document the statistical approach we used and identify design challenges and strengths.

The design we propose takes advantage of the comparison of pre- to post-implementation within sites along with comparisons between QII implementation and usual care sites found in the standard stepped wedge design, thus maximizing the utilization of available data. In the nonrandomized design, however, the actual timing of implementation is incorporated into the statistical assessment of intervention effectiveness. The design accounts for potential threats to validity due to historical trends [8] by incorporating data from sites that have started exposure to the intervention (are “on”) as well as data from sites that have not yet been exposed (are “off”) at any time point.

In addition to accounting accurately for the timing of an intervention, the nonrandomized design is particularly suited to the needs of managers and policymakers in multilevel, data rich managed care organizations. These organizations often evaluate potential policy or practice improvement strategies in selected sites before crafting an organization-wide implementation approach. The nonrandomized design we describe accounts accurately for the level of QII implementation; in the case we present, the level of implementation (and clustering) is at the primary care practice. The design can also integrate the full range of relevant data collected by the organization through its routine activities. In the case we discuss, we use a standard organizational survey as an outcome, and account for context based on available administrative data.

Evaluation models such as “difference in differences” [9] or mixed models with repeated measures are similar to the nonrandomized stepped wedge design presented in this paper, and may be more appropriate if there are relatively few implementation time frames. Our previous work with a repeated measures model included one pre-implementation and two post-implementation periods [10]. The simplest form of a difference-in-differences approach involves two time frames: before and after. While a difference-in-difference approach can accommodate more than two time frames, it can become unwieldy as additional time points are incorporated because it requires specification of indicator variables for each time period and their interaction with treatment groups. Moreover, a difference-in-differences approach may lose both precision and information if the time frames over which sites implement the QII are lengthy (such as in our example QII study with 12 time periods when QII implementation may take place) because the approach requires collapsing the time intervals to obtain pre- and post-implementation means with a concomitant loss of information.

Simple pre-post designs, particularly when using enhanced quality improvement statistical methods [11, 12], are an alternative to randomized designs. Yet these types of designs are unlikely to be successful when there are strong period effects. In the example QII studied here, we expect strong historical trends given that the QII is being carried out within an overall change in the organization’s primary care model. Thus, successful evaluation of the studied QII will require a rigorous comparison group that can establish whether or not the intervention has effects over time above those produced by the overall organizational changes.

As is true in randomized stepped wedge designs, we will introduce the QII such that a site that had no intervention initially, but adopted the QII later, can serve as a comparator to both itself and to implementation states for other sites. This latter feature enables the fullest possible use of data from all sites by using control state and implementation state data from each site, enhancing validity.

In summary, to evaluate our planned QII, key design features we considered included the need to: 1) use data collected in an ongoing fashion before and after implementation of the intervention in all study sites; 2) account for multiple levels of observation (patient and site across time); and 3) include accurate information on the timing of the QII implementation so that respondents within sites exposed to control or implementation states can be assessed at the same time points.

While the nonrandomized stepped wedge design can be understood intuitively, the underlying statistical model is more complex. Through the work presented here, we aimed to both prospectively solve analytic issues with the design. We also aimed to test the feasibility of the design for detecting an optimistic but realistic effect size when applied to evaluation data simulated to reflect the characteristics of actual data available for assessing the planned QII and hope this work will encourage discussion between investigators and statisticians.

## Methods

### Study Setting

The QII we simulated aims to enhance implementation of the Patient-Centered Medical Home (PCMH), an approach to primary care in VA primary care practices. The PCMH model, termed Patient Aligned Care Teams (PACT) in VA’s application, began national implementation across the VA system in 2010. The QII is an add-on to the PACT implementation within selected primary care practice sites. It focuses on a researcher/clinical leader partnership aimed at enhancing the rapidity and completeness of PACT implementation. The evaluation outcome variable we simulated is patient-assessed access to care. This variable was simulated to reflect data from a patient experience survey administered by VA to randomly selected patients within care sites in an ongoing sampling process over time.

### QII Implementation Timing

**Fig. 1:**
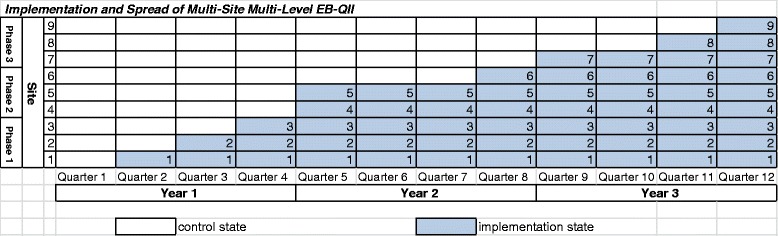
Implementation and spread of multisite multilevel Qll

We simulated data for a multilevel, multisite (*n* = 9) QII. The roll out of the intervention was nonrandom and nonsystematic: all sites started out at Quarter 1 in the control state. There were three phases, where each phase consisted of sites implementing the QII closely together, although not simultaneously due to the logistical constraints sites faced. From an administrative standpoint, we judged a phase to be one fiscal year when our team engaged with sites to implement the QII. The three Phase 1 sites (Sites 1, 2 and 3) implemented the QII in Quarters 2, 3, and 4, respectively. Two of the three Phase 2 sites (Sites 4 and 5), implemented the QII in Quarter 5, and the other Phase 2 site (Site 6) implemented the QII in Quarter 8. Among Phase 3 sites, Site 7 implemented in Quarter 9, and Sites 8 and 9 implemented in Quarters 11 and 12, respectively (Fig. 1). In this allocation scheme, we assumed that the data collection and assessment of the QII occurred in each of the 12 quarters.

### Study Sample

**Fig. 2:**
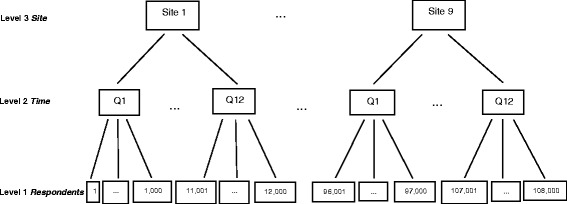
Multilevel structure of repeated cross-sectional design in example QII

The sample of simulated data consisted of a total of 108,000 respondents to patient experience surveys over 12 quarters at nine sites. A different cross-section of 1000 patients was sampled at each site and each quarter. Figure 2 illustrates, for example, that respondents 1 to 1000 were surveyed in Quarter 1 at Site 1, and respondents 107,001 to 108,000 were surveyed in Quarter 12 at Site 9.

### Measures

Improving access to care for enrolled patients is a key goal for many primary care practice improvement efforts, including the QII under consideration here. In addition, the QII has goals of improving continuity and coordination of care in PCMH implementation. We chose to focus on access as an outcome in our simulated data because it is a process-of-care measure that is tracked closely and questions pertaining to access are asked in patient surveys at VA. Moreover, we chose to simulate access because we view it as a more proximal process-of-care than are continuity, coordination of care, or other measures included in the survey.

Access to care is reflected in the degree to which patients are able to, for example, call and make an appointment as soon as they need it. We used the Likert scale access question: “How often did you receive care as soon as you needed it during the last 12 months (access)”. Respondents answered “never”, “sometimes”, “usually”, or “always”. Since respondents are Level 1 units, we designated access as measured by this question as a Level 1 variable.

We simulated the outcome (access) as a random variable with a uniform distribution for sites in the control state. Thus, 25 % of respondents answered they “always” got care as soon as needed in the past 12 months, 25 % “usually”, 25 % “sometimes” and 25 % “never” had access. Our choice of a uniform distribution reflects preliminary data from observed patient surveys. For sites in the implementation state, we simulated the outcome as having marked improvement immediately after implementing the QII so that the percentages of patients answering “always” having access immediately after implementation of the QII ranged between 30 and 36 %, while percentages with “usually” ranged from 29 and 33 %. The new distribution represented an improvement of 4 to 11 % compared to control. Conversely, percentages of patients with “sometimes” access after implementation decreased to 16 and 23 %, and “never” decreased to 11 and 20 %. While sites continued to implement the QII over time, we simulated continued improvement in the outcome, but with a slower rate of improvement, ranging from one to three percent in each ensuing quarter, such that that the percentages of patients with “always” ranged from 31 to 44 %, “usually” from 30 to 42 %, “sometimes” from 7 to 22 %, and “never” from 5 to 18 %.

The basis for the improvement when sites were exposed to implementation was based on prior work, where QIIs were implemented using the Plan-Do-Study-Act cycles and continuous improvement was expected. In addition to accommodating the scenario described here, we believe the analytic strategy and statistical model presented below are flexible enough to accommodate many possible trends in outcome over time (for example, if patient-assessed access improved at a constant rate, have slow improvement at the beginning of implementation, remained stagnant or even decreased).

One of the main predictors in the model was time (Quarter 1 to Quarter 12). In the context of the simulated data, this was the linear time trend during which the organization was involved in an overall change in the primary care model. The second predictor was treatment status (“on” or “off”) that indicates when the sites were exposed to the implementation state or control state, respectively (Fig. 1). In addition, we represented the difference in linear time trend for sites before and after implementation of the QII as a time-by-treatment interaction, which was included as another independent variable.

Our main predictors thus occupy Level 2 of the hierarchical modeling [13], because it incorporated the time trend between pre- and post-implementation of the QII. To ensure maximum comparability across sites in the outcome measure, we also simulated covariates at Level 1 (respondents) and Level 3 (sites). Specifically, we simulated gender and race/ethnicity at Level 1, and site size at Level 3. We simulated gender and race/ethnicity as dichotomous random variables with 90 % of the respondents males and 75 % whites as in the VA study sites. We simulated site size as a random variable with values of “small”, “medium”, or “large.” In our data, two sites were small, two sites medium, and five sites large.

Respondent-level and site-characteristics were not weighted. While we included respondent-level and site-level characteristics in our simulated data, we did not simulate a differential effect on patient-assessed access for these variables in our analyses. We did not have an a priori hypothesis that the QII would affect women, minorities or respondents from large sites differently than others. Our main purpose in including race/ethnicity, gender and site size in the analysis was to ascertain whether the nonrandomized stepped wedge analytic design can feasibly accommodate patient-level and site-level characteristics that might be associated with actual timing of QII implementation.

Having simulated the data in one data set, we then used the nonrandomized stepped wedge design to evaluate the QII. We built into the simulation the time trend we expected i.e., sites had 4 to 11 % improvement in their first quarter of implementation, and 1 to 3 % in each ensuing quarter thereafter when sites continued to implement the QII, and no improvement based on historical trends seen in PACT. We considered our analytic approach to be successful if our nonrandomized stepped wedge analysis confirms the trends we simulated in the data.

### Analytic Strategy

The simulated data was multilevel: while the intervention was targeted at the site level (thus minimizing contamination), where a site was either in the control or implementation state, the outcome of interest was measured at the individual patient level. Moreover, time (when the intervention was implemented) constituted another level in the study design. In all, there were three levels: individual patients, time of implementation, and sites.

The first analytic decision we confronted was which levels of clustering to address. Multilevel models cannot validly integrate more levels than the data can support. In our case, accounting for clustering of patients within providers, sites, and time was not feasible. Linkage between providers and patients was not stable over time, and inclusion of four levels resulted in over-specification in the model-fitting algorithm. Furthermore, other analyses of the survey showed that site-level clustering was stronger than provider-level clustering. We therefore decided on a three-level hierarchical data structure in the form of a repeated cross-sectional design with multiple layers of clustering [14]: patients within time and within site (primary care practice), and in turn, time clustered within site [15–17]. In the final analysis structure, respondent patients were the Level 1 units, the times at which the patients were surveyed were the Level 2 units, and sites themselves were the Level 3 units (Fig. 2).

Our analytic strategy accounted for timed outcome assessments at the patient level, random effects on outcomes at the site level, and the time period during which the QII was implemented at individual sites. The estimation method in the HLM software is equivalent to generalized estimation equation (GEE) because the outcome of interest was ordinal and has been known to be more robust to misspecification of the variance structure [18].

#### Statistical model for a nonrandomized stepped wedge design

We represented our nonrandomized stepped wedge design using a statistical model with a three-level hierarchical data structure. The patient-assessed access was a four category ordinal outcome (1 = always, 2 = usually, 3 = sometimes and 4 = never). The parameter patient-assessed access (*Y*_*ijk*_) represented the outcome determined on the *i* ^*th*^ respondent in the *j* ^*th*^ time point (quarter) from the *k* ^*th*^ site. Moreover, *Y*_.*jk*_ was the site-level mean outcome of interest in the j^th^ quarter and the k^th^ site. The site-level mean was obtained by summing access scores from the respondents from each quarter at each site and then dividing by the number of respondents (1000 in this case).

The main model predictors were at Level 2, where *TREAT*_*jk*_ was the indicator (1=“on”; 0=“off”) for treatment status for the *j* ^*th*^ site in the *j* ^*th*^ quarter. The linear time trend when sites were in the control state was represented by *QTR*_*jk*_ (*k* ^*th*^ site in *j* ^*th*^ quarter), and linear time trend when sites were exposed to the implementation state was represented by *QTR*_*jk*_ * *TREAT*_*jk*_ for (*k* ^*th*^ site in *j* ^*th*^ quarter). This parameter represented actual time of implementation. At the respondent level, *FEMALE*_*ijk*_ was a covariate for gender. *NONWHITE*_*ijk*_ was a second respondent-level covariate. At the site-level, we had two indicator variables to represent size: *MED*_*k*_ (=1 if *k* ^*th*^ site was a medium size site, 0 if not) and *LG*_*k*_ (=1 if *k* ^*th*^ site was a size large, 0 if not).

The response options were ordinal, and thus regressions were defined as cumulative logits. Moreover, because there were four categories in the response options, there were three cumulative splits [19], where the response options were categorized as: 1) “always” versus “usually”, “sometimes”, or “never”; 2) “always” or “usually” versus “sometimes” or “never”; and 3) “always”, “usually”, or “sometimes” versus “never.”

The statistical model relating access and predictors at all three levels were formulated as the following expressions:

1 $$ \log \left[\frac{\varnothing_{1ijk}^{*}}{1-{\varnothing}_{1ijk}^{*}}\right] = {\gamma}_{000}+{\gamma}_{010}QT{R}_{jk}+{\gamma}_{020} TREA{T}_{jk}+{\gamma}_{030}QT{R}_{jk}* TREA{T}_{jk}+{\gamma}_{100} FEMAL{E}_{ijk} + {\gamma}_{200} NONWHIT{E}_{ijk} + {\gamma}_{001} ME{D}_k+{\gamma}_{002}L{G}_k+{r}_{0jk}+{u}_{00k} $$

2 $$ \log \left[\frac{\varnothing_{2ijk}^{*}}{1-{\varnothing}_{2ijk}^{*}}\right] = {\gamma}_{000}+{\gamma}_{010}QT{R}_{jk}+{\gamma}_{020} TREA{T}_{jk}+{\gamma}_{030}QT{R}_{jk}* TREA{T}_{jk}+{\gamma}_{100} FEMAL{E}_{ijk} + {\gamma}_{200} NONWHIT{E}_{ijk} + {\gamma}_{001} ME{D}_k+{\gamma}_{002}L{G}_k+{r}_{0jk}+{u}_{00k}+{\delta}_2 $$

3 $$ \log \left[\frac{\varnothing_{3ijk}^{*}}{1-{\varnothing}_{3ijk}^{*}}\right] = {\gamma}_{000}+{\gamma}_{010}QT{R}_{jk}+{\gamma}_{020} TREA{T}_{jk}+{\gamma}_{030}QT{R}_{jk}* TREA{T}_{jk}+{\gamma}_{100} FEMAL{E}_{ijk} + {\gamma}_{200} NONWHIT{E}_{ijk} + {\gamma}_{001} ME{D}_k+{\gamma}_{002}L{G}_k+{r}_{0jk}+{u}_{00k}+{\delta}_3 $$

Thus, Eq. (1) was the log-odds of respondents answering “always” relative to “usually”, “sometimes” or “never” having access, while Eq. (2) was the log-odds of respondents answering “always” or “usually” relative to “sometimes” or “never” having access, and Eq. (3) was the log-odds of respondents answering “always”, “usually”, or “sometimes” relative to “never” having access. The parameters *r*_0*jk*_ and *u*_00*k*_ represented the random effects associated with time-level and site-level random effects, respectively. The parameters *δ*_2_ and *δ*_3_ were the thresholds that separated their respective cumulative logits. Appendix 1 provides a more detailed treatment of the statistical model of our analysis.

All data construction, simulation, and diagnostics were conducted in SAS 9.3 (SAS Institute Inc., Cary, NC, USA). Data analysis and modeling were performed using the HLM 7 software (Scientific Software International Inc., Skokie, IL, USA). The HLM software enabled us to model a repeated cross-sectional three-level hierarchical model with an ordinal outcome.

## Results

**Fig. 3:**
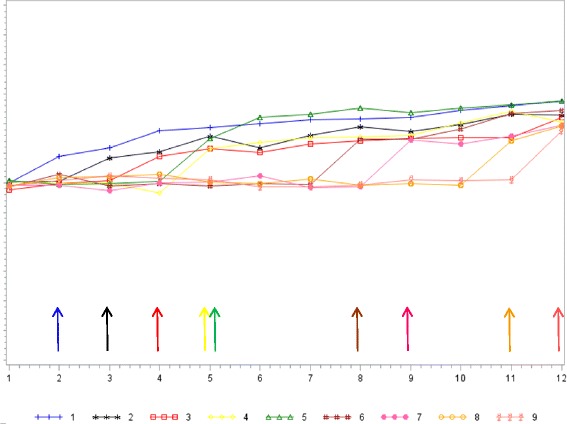
Mean simulated patient-assessed access for sites over time

The mean site scores for patient-assessed access over time were shown in Fig. 3. All sites were in control state at Quarter 1. Following Quarter 1, sites had no improvement in access while in the control state. However, sites had marked improvement immediately after implementing the QII and access continued to improve thereafter, albeit at a slower rate.

The results for the multivariate analysis of the main predictors, and individual and site characteristics are presented in Table 1.

**Table 1:** Results for the three-level ordinal model with timing of implementation, linear time trends, respondent-level, and site-level characteristics

Parameter	Model 1	Model 2	Model 3	Model 4
	Coeff (SE)/OR	Coeff (SE)/OR	Coeff (SE)/OR	Coeff (SE)/OR
Intercept, *γ*_000_	−1.22 (0.04)/0.29**	−0.821 (0.074)/0.440**	−0.874 (0.144)/0.417**	−1.235 (0.063)/0.291**
Threshold 2, *δ*_2_	1.294 (0.006)/3.647**	1.294 (0.006)/3.647**	1.294 (0.006)/3.647**	1.294 (0.006)/3.647**
Threshold 3, *δ*_3_	2.345 (0.009)/10.437**	2.345 (0.009)/10.437**	2.345 (0.009)/10.437**	2.345 (0.009)/10.437**
Time-level predictors				
QTR, *γ*_010_	0.005 (0.005)/1.005			0.005 (0.005)/1.005
TREAT, *γ*_020_	0.265 (0.048)/1.303**			0.262 (0.048)/1.300**
QTR^a^TREAT, *γ*_030_	0.049 (0.007)/1.051**			0.049 (0.007)/1.051**
Respondent-level predictors				
Gender (ref: MALE)				
FEMALE, *γ*_100_		−0.007 (0.018)/0.993		−0.007 (0.018)/0.993
Race/ethnicity (ref: WHITE)				
NONWHITE, *γ*_200_		−0.013 (0.013)/0.987		−0.013 (0.013)/0.988
Site-level predictors				
Site Size (ref: SMALL)				
MED, *γ*_001_			−0.085 (0.204)/0.918	−0.040 (0.083)/0.961
LG, *γ*_002_			0.122 (0.171)/1.130	0.049 (0.069)/1.050

**p < 0.001

*Note*: ^a^ < 0.05

In each of the models, the intercept parameter, *γ*_000_, was the cumulative logit of “always” getting care as soon as needed relative to “usually”, “sometimes”, and “never”, adjusting for the predictors in the model. The sum of *γ*_000_ and *δ*_2_ represented the cumulative logit of “always” or “usually” relative to “sometimes” or “never” getting care. Likewise, the sum of *γ*_000_ and *δ*_3_ was the cumulative logit of “always”, “usually”, or “sometimes” relative to “never” getting care. In the models, the value of *γ*_000_ was negative, meaning that there was a low likelihood of “always” getting care as soon as needed. The sums of *γ*_000_ and *δ*_2_, and *γ*_000_ and *δ*_3_ were both positive and increasing, meaning that the likelihood steadily increased across the response options (i.e., from “always” to “usually” to “sometimes” access).

Model 1 contained the timing-related predictors, namely, status of when sites were exposed to implementation (*TREAT*_*jk*_), linear time trends in the control state (*QTR*_*jk*_), and implementation state (*QTR*_*jk*_ * *TREAT*_*jk*_). The cumulative logit coefficient for treatment main effect, *γ*_020_, was 0.265. Transforming the coefficient into (OR = *e* ^0.265^ = 1.303) suggested that when sites were exposed to the implementation, they had about a 30 % increase in the odds of having improved access in the previous 12 months, i.e., compared to respondents in the control state, respondents within sites that were in the implementation state were more likely to change their responses from “usually” to “always”, “sometimes” to “usually”, or “never” to “sometimes.” The treatment main effect parameter was significant (*p* < 0.001).

Linear time trends in Model 1 were represented by two fixed effects: *γ*_010_ for when sites were in the control state, *QTR*_*jk*_, and *γ*_030_ for when sites were in the implementation state, *QTR*_*jk*_ * *TREAT*_*jk*_. The *γ*_010_ parameter was not significant (*p* > 0.05): there were no changes in respondents’ answers in getting care as soon as needed in each ensuing quarter while sites were in the control state. However, in each ensuing quarter when sites were in the implementation state (*γ*_030_) was associated with about a 5 % increase in the odds of improvement in getting care as soon as needed in the previous 12 months (*p* < 0.001).

Model 2 contained respondent-level predictors, namely, *FEMALE*_*ijk*_ and *NONWHITE*_*ijk*_. The parameter *γ*_100_ was not statistically significant, meaning that female respondents did not differ from their male counterparts in their likelihood of patient-assessed access. Similarly, *γ*_200_ was not statistically significant and thus, respondents who were of non-white race/ethnicity did not differ from their white counterparts in their likelihood of patient-assessed access.

Model 3 contained site-level predictors: *MED*_*k*_, an indicator for medium size sites, and *LG*_*k*_, an indicator for large size sites. Compared to small size sites, respondents from medium size (*γ*_001_) and large size (*γ*_002_) sites were not statistically different in their likelihood of access.

Lastly, Model 4 was the full model, comprising of time-level, respondent-level, and site-level predictors. The results were similar to those found in the previous models.

## Discussion

We demonstrated the feasibility of applying a nonrandomized stepped wedge design to patient-level assessments of access as an outcome of a site-level QII, using simulated data as a precursor to applying the design to actual data that will use the same variables. The results of the nonrandomized stepped wedge design were consistent with the effects we simulated in the data. In the control state at Quarter 1, there was a low likelihood of having access to care. Once sites entered their first quarter of implementation, we simulated the data so that there was an average of about 30 % increased odds of improvement in access. As sites continued with implementation, the results showed that our statistical approach detected the expected approximate 5 % increased odds of improvement in the outcome of interest in each time quarter due to the QII. Thus, the analytical model demonstrated significant improvement in access as sites crossed over from control to implementation states, and also when sites remained in implementation in subsequent quarters. We should note that our results might be biasing towards the null because data collection occurred every quarter while the survey question asked respondents about their experience in the past 12 months, which might include time periods in which sites have not yet implemented the QII.

Our simulation included many of the design elements that may make evaluating QIIs a challenging exercise. Specifically, we simulated data from a three-level hierarchical repeated cross-sectional design whereby respondents (Level 1) were clustered within time points (Level 2), which in turn were clustered within sites (Level 3). The timing of a site’s initiation of the QII was nonrandom, the outcome of interest had an ordinal distribution, and the main predictors of interest were time points, treatment status, and the interaction of time points and treatment status.

This exploratory analysis showed that while these issues might not be unique to the nonrandomized stepped wedge design, managers and policy makers in data rich organizations wishing to use the nonrandomized stepped wedge design should still be cognizant that the final evaluation of QII using routinely collected data will need to meet a number of requirements. First, the study’s evaluation database will need to be constructed as a three-level database that can link patients to their sites, and their sites to intervention time periods and timing of the data collected. Constructing this database, therefore, will require accurate collection of data on the time at which sites “turned on,” i.e. implemented the QII. It will also require multiple cross-sectional data collection of the outcome variable (in this case, patient-assessed access), and inclusion of accurate dating of the outcome variable. Finally, it will require a computer program that can handle large amounts of multilevel data efficiently within multivariable models. In our case, this required shifting from SAS to HLM 7. Achieving a successful analytic model, even with simulated data, proved challenging and required a variety of problem-solving efforts. Thus, while nonrandomized stepped wedge designs are appealing for evaluating QII’s, substantial care and expertise are required to actually analyze data within the design.

To our knowledge, this paper represents the first application of a nonrandomized stepped wedge design to evaluate implementation of a QII. Our approach may be helpful in improving evaluation accuracy under this circumstance. In addition, although we plan to use the design prospectively, it would be possible to apply it retrospectively if accurate data on intervention timing and on outcomes are available.

In the future, we plan on taking up analyses using difference-in-differences or pre- versus post-implementation approaches in addition to using the nonrandomized stepped wedge design as our survey data become available. This would allow us to compare outcomes using various analysis techniques.

These exploratory data analyses were limited in a variety of respects. We assumed each cross-section was independent; in reality, while patients were simulated to be randomly selected to receive the survey from among all those visiting each site, some patients may be sampled more than once over time. In addition, we assumed a fairly large effect size; yet a smaller effect size could still have clinical significance in an actual evaluation. We also assumed continued improvement in the outcome over time, although at a diminishing level; this was in line with our aim of testing a realistic “best case’ for improvement. Third, we made the choice of using site random effects. Our study intervention is a quality improvement initiative; as is often the case in quality improvement, the assumption that our observed effect size represents a random sample of the possible effect sizes that could have been observed may be most appropriate for estimating future impacts of intervention spread. Lastly, we made the choice of using site random effects. Our study intervention is a quality improvement initiative; as is often the case in quality improvement, the assumption that our observed effect size represents a random sample of the possible effect sizes that could have been observed may be most appropriate for estimating future impacts of intervention spread. Lastly, we made the choice to report the results in terms of means and did not transform the access variable into dichotomous splits because we didn’t know the meaningful separation between the response options.

## Conclusion

The design features of the actual QII that were the basis for this simulation, as well as the outcome measure used, imposed a variety of constraints on other analytic approaches that we could have used to evaluate the QII such as difference-in-differences or repeated measures approaches. These design features included nonrandomized timing of the QII, clustering of the outcome within sites as sites were repeatedly measured, and successive implementation in which sites that were originally control sites “turned on” to become implementation sites. Our application of the nonrandomized stepped wedge design matches well to the evaluation needs of this complex multisite QII. It addresses issues of contamination (by having the QII target sites, and aiming to expose all respondents within sites in the implementation state to the QII) and historical threats (by having data collected from sites in the control and implementation states at each quarter), all the while allowing for variable and nonrandom timing in implementing the intervention. It also allows for a wide time frame without losing precision of information about intervention effects.

Healthcare evaluators have long struggled with the challenges posed by the need to evaluate QIIs implemented primarily by a healthcare organization, with limited researcher control. The marked increases in data collected by these organizations for routine quality monitoring or improvement provide an outstanding opportunity to apply rigorous designs such as the nonrandomized stepped wedge that substantially avoid the typical validity threats to simple pre-post designs. We expect our work will encourage other QII researchers to consider and further develop the nonrandomized stepped wedge design as they confront the challenge of evaluating QII implementation and spread within healthcare organizations.

## Appendix 1: Three-level Hierarchical Statistical Model of the Nonrandomized Stepped Wedge Design

### Level 1 Model: Relationship between Patient-assessed Access and Patient-level Covariates

The statistical equations at Level 1 expressed the relationship between the outcome of interest, patient-assessed access (*Y*_*ijk*_) and respondent-level covariates, namely, gender (*FEMALE*_*ijk*_) and race/ethnicity (*NONWHITE*_*ijk*_). For the variable *FEMALE*_*ijk*_, male gender served as the reference, and for the variable *NONWHITE*_*ijk*_, white race/ethnicity served as the reference.

Access was a four-level ordinal outcome, and thus, was represented by three cumulative logits.

Equation (R.1) expressed the log-odds of “always” relative to “usually”, “sometimes”, or “never” having access:

R.1 $$ \log \left[\frac{\varnothing_{1ijk}^{*}}{1-{\varnothing}_{1ijk}^{*}}\right]={\pi}_{0jk}+{\pi}_{1jk} FEMAL{E}_{ijk} + {\pi}_{2jk} NONWHIT{E}_{ijk} $$

Equation (R.2) expressed the log-odds of “always” or “usually” relative to “sometimes” or “never” having access. The parameter *δ*_2_ was the threshold parameter that split the cumulative logits.

R.2 $$ \log \left[\frac{\varnothing_{2ijk}^{*}}{1-{2}_{2ijk}^{*}}\right]={\pi}_{0jk}+{\pi}_{1jk} FEMAL{E}_{ijk} + {\pi}_{2jk} NONWHIT{E}_{ijk}+{\delta}_2 $$

Lastly, Eq. (R.3) expressed the log-odds of “always”, “usually”, or “sometimes” having access relative to “never” having access. Likewise, the parameter *δ*_3_ was the threshold parameter that split the cumulative logit (R.3) from the other cumulative logits (R.1 & R.2).

R.3 $$ \log \left[\frac{\varnothing_{3ijk}^{*}}{1-{3}_{2ijk}^{*}}\right]={\pi}_{0jk}+{\pi}_{1jk} FEMAL{E}_{ijk} + {\pi}_{2jk} NONWHIT{E}_{ijk}+{\delta}_3 $$

### Level 2 Model: Linear Time Trend in Control versus Implementation State

Equations at Level 2 expressed the relationships between respondent-level covariates (which served as Level 2 outcomes) and timing-related predictors, including treatment status (*TREAT*_*jk*_), and linear time trends when sites were in the control state (*QTR*_*jk*_) and when they were in implementation state (*QTR*_*jk*_ * *TREAT*_*jk*_). The parameter *r*_0*jk*_ was the timing-level random effects.

T.1 $$ {\pi}_{0jk} = {\beta}_{00k}+{\beta}_{01k}QT{R}_{jk}+{\beta}_{02k} TREA{T}_{jk}+{\beta}_{03k}QT{R}_{jk}* TREA{T}_{jk}+{r}_{0jk} $$

T.2 $$ {\pi}_{1jk}={\beta}_{10k} $$

T.3 $$ {\pi}_{2jk}={\beta}_{20k} $$

### Level 3 Model: Site-level Effects

Equations at Level 3 expressed the relationships between timing-level variables and site-level predictors, namely, site size (*MED*_*k*_ and *LG*_*k*_). Please note that with indicator variables for medium and large size sites in the models, small size sites served as the reference. The parameter *μ*_00*k*_ represented the site-level random effect.

S.1 $$ {\beta}_{00k}={\gamma}_{000}+{\gamma}_{001} ME{D}_k+{\gamma}_{002}L{G}_k+{\mu}_{00k} $$

S.2 $$ {\beta}_{01k}={\gamma}_{010} $$

S.3 $$ {\beta}_{02k}={\gamma}_{020} $$

S.4 $$ {\beta}_{03k}={\gamma}_{030} $$

S.5 $$ {\beta}_{10k}={\gamma}_{100} $$

S.6 $$ {\beta}_{20k}={\gamma}_{200} $$

### Mixed Linear Models

All equations at the three-level can be combined and expressed as the following cumulative logits:

Log-odds of respondents “always” relative to “usually”, “sometimes”, or “never” having access -

4 $$ \log \left[\frac{\varnothing_{1ijk}^{*}}{1-{\varnothing}_{1ijk}^{*}}\right] = {\gamma}_{000}+{\gamma}_{010}QT{R}_{jk}+{\gamma}_{020} TREA{T}_{jk}+{\gamma}_{030}QT{R}_{jk}* TREA{T}_{jk}+{\gamma}_{100} FEMAL{E}_{ijk} + {\gamma}_{200} NONWHIT{E}_{ijk} + {\gamma}_{001} ME{D}_k+{\gamma}_{002}L{G}_k+{r}_{0jk}+{u}_{00k} $$

Log-odds of respondents “always” or “usually” relative to “sometimes” or “never” having access -

5 $$ \log \left[\frac{\varnothing_{2ijk}^{*}}{1-{\varnothing}_{2ijk}^{*}}\right] = {\gamma}_{000}+{\gamma}_{010}QT{R}_{jk}+{\gamma}_{020} TREA{T}_{jk}+{\gamma}_{030}QT{R}_{jk}* TREA{T}_{jk}+{\gamma}_{100} FEMAL{E}_{ijk} + {\gamma}_{200} NONWHIT{E}_{ijk} + {\gamma}_{001} ME{D}_k+{\gamma}_{001}L{G}_k+{r}_{0jk}+{u}_{00k}+{\delta}_2 $$

Log-odds of respondents “always”, “usually”, or “sometimes” relative to “never” having access -

6 $$ \log \left[\frac{\varnothing_{3ijk}^{*}}{1-{\varnothing}_{3ijk}^{*}}\right] = {\gamma}_{000}+{\gamma}_{010}QT{R}_{jk}+{\gamma}_{020} TREA{T}_{jk}+{\gamma}_{030}QT{R}_{jk}* TREA{T}_{jk}+{\gamma}_{100} FEMAL{E}_{ijk} + {\gamma}_{200} NONWHIT{E}_{ijk} + {\gamma}_{001} ME{D}_k+{\gamma}_{001}L{G}_k+{r}_{0jk}+{u}_{00k}+{\delta}_3 $$

Note that Eqs. (4), (5), and (6) can be combined into a single generic expression:

$$ \begin{array}{l}{\eta}_{mijk}= \log \left[\frac{\varnothing_{mijk}^{*}}{1 - {\varnothing}_{mijk}^{*}}\right]\\ {}\kern6em  = {\gamma}_{000}+{\gamma}_{010}QT{R}_{jk}+{\gamma}_{020} TREA{T}_{jk}+{\gamma}_{030}QT{R}_{jk}* TREA{T}_{jk}+{\gamma}_{100} FEMAL{E}_{ijk}\\ {}\kern6em +{\gamma}_{200} NONWHIT{E}_{ijk}+{\gamma}_{001} ME{D}_K+{\gamma}_{002}L{G}_k+{r}_{0jk}+{u}_{00k}+{\displaystyle \sum_{m=2}^{M-1}}{D}_{mijk}{\delta}_m\end{array} $$

## Abbreviations

CSHIIP: Study of healthcare innovation, implementation & policy
EDM: Electric data methods
HSR&D: Health services research and development
PACT: Patient aligned care teams
PCMH: Patient-centered medical home
QII: Quality improvement interventions
UCLA: University of California at Los Angeles
VA: Veterans Health Administration
